# Home injuries and built form – methodological issues and developments in database linkage

**DOI:** 10.1186/1472-6963-5-12

**Published:** 2005-02-02

**Authors:** Robert G Newcombe, Ronan A Lyons, Sarah J Jones, Joanne Patterson

**Affiliations:** 1 Department of Epidemiology, Statistics and Public Health, Cardiff University, Heath Park, Cardiff CF14 4XN, UK; 2 The Clinical School, University of Wales Swansea, Grove Building, Singleton Park, Swansea SA2 8PP, UK; 3 Welsh School of Architecture, Cardiff University, Bute Building, King Edward VII Avenue, Cardiff, CF10 3NB, UK

## Abstract

**Background:**

The aim of this body of research is to determine whether injuries in the home are more common in particular types of housing. Previous home injuries research has tended to focus on behaviours or the provision of safety equipment to families with young children. There has been little consideration of the physical environment. This study reports methodological developments in database linkage and analysis to improve researchers abilities to utilise large administrative and clinical databases to carry out health and health services research.

**Methods:**

The study involved linking a database of home injuries obtained from an emergency department surveillance system with an external survey of all homes in an area and population denominators for home types derived from a health service administrative database. Analysis of injury incidence by housing type was adjusted for potential biases due to deprivation and distance to hospital. For non-injured individuals data confidentiality considerations required the deprivation and distance measures be imputed. The process of randomly imputing these variables and the testing of the validity of this approach is detailed.

**Results:**

There were 14,081 first injuries in 112,248 residents living in 54,081 homes over a two-year period. The imputation method worked well with imputed and observed measures in the injured group being very similar. Re-randomisation and a repeated analysis gave identical results to the first analysis. One particular housing type had a substantially elevated odds ratio for injury occurrence, OR = 2.07 (95% CI: 1.87 to 2.30).

**Conclusions:**

The method of data linkage, imputation and statistical analysis used provides a basis for improved analysis of database linkage studies.

## Background

Home injuries are frequent and result in greater mortality and morbidity than road traffic injuries[1]. It is evident that the risks of some types of injuries, such as falls or injuries resulting from fire, could well be related to the built form of the home. Nevertheless the availability of robust evidence based on large numbers of cases is quite limited[2]. Previous home injuries research has tended to focus on behaviours or the provision of safety equipment to families with young children. There has been little consideration of the physical environment. Linkage of existing large datasets has the potential to yield substantial evidence.

The present study was designed to utilise three such datasets relating to all residential properties in a defined geographical area in the United Kingdom, the resident population, and their attendances at local hospital emergency departments (EDs). These datasets could not be linked comprehensively at individual level on account of constraints on identifiability of individuals. This article describes the novel challenges that result, and the methodology used to obviate them.

## Methods

This study was carried out as part of the wider Housing and Neighbourhood and Health (HANAH) project[3]. This is a long-term partnership between academia and local authorities to elucidate the relationship between the social and built environment and health and to develop interventions to improve health. Data from an injury surveillance system on injury events in residents of the Neath-Port Talbot County Borough Council area was linked to a register of property types and denominator data. Ethical permission for the study was granted by the Morgannwg Local Research Ethics Committee.

All properties in the study area were viewed externally. Domestic properties were classified into categories based on floor area (four groups), five period groups, and five build types, viz. detached, semi-detached, flat conversions, purpose-built flats, and terraced housing. Ninety-four of the 100 combinations of the three housing type variables were found in the study area. Analysis was carried out at individual property level: analysis at postcode or zip code level was not possible because very few postcodes (13%) comprised a single property type. Postcodes contained an average of 14 properties.

Data on injuries treated at EDs were obtained for the period 1999 – 2000 from the All Wales Injury Surveillance System (AWISS) which routinely obtains individual level data from EDs surrounding the study area and is described in detail elsewhere[4]. Briefly, the data comprises the patient's address, age, sex, date of occurrence, type and anatomical site of injury (up to three diagnoses and three sites can be coded), and includes a code indicating whether the injury occurred at home or not. No information on precipitating factors such as falls, fires or drug abuse is included.

To obtain denominator data for each property type we used the National Health Service Administrative Register (NHSAR), a list of all people registered with the free-to-use primary care health service in Wales. Data on this system are highly confidential and denominator population profiles were obtained by providing a list of all properties in the ninety four different groups to the NHSAR staff who then matched these with their system and obtained the number of people in each of the property types, subdivided by age and sex. This system has previously been used to obtain small area population data[5].

In analysing data on injury attendance at hospital EDs it is important to take into account the potential confounders of deprivation and access, which are known to be strongly related to injury occurrence and ED attendance respectively[6]. For each of the injured individuals it was possible to assign an exact value for the Townsend Index of Material Deprivation and distance to hospital by road as the individual addresses were available[7]. The Townsend Index is a small area based deprivation index, commonly used in epidemiological studies in the UK, and derived from four census variables: home ownership, overcrowding, access to a car, and unemployment. It has been shown to be strongly related to the incidence of specific types of injuries[5,6]. For non-injured individuals this was not possible due to confidentiality constraints described above – only data aggregated at groups of address level was available. Linkage had to be performed in an indirect manner because a small number of properties in a single electoral division meant that data on age/sex compilation could be considered potentially identifiable and so could not be released.

During 1999 and 2000, 14,171 out of 112,248 residents made one or more emergency department visits for a home injury. We sought to combine three files comprising individual-level injuries data from an emergency department surveillance system; an external assessment of the built form in all 54,801 homes in the area; and denominator populations for each of ninety-four property types delivered from a health service registration system. Application of logistic regression to model injury risk on built form, property size and age, subject age, sex, deprivation and distance from emergency department jointly necessitates construction of a single linked data file. The objective was to construct an appropriately linked database and hence determine whether injuries occur more commonly in different types of home.

The study population of 112,248 individuals made 16,358 ED attendances for home injuries during the study period. The vast majority (99.5%, n = 16,277) of these attendance records included adequate data on age, gender, proximity and Townsend score. These 16,277 attendances involved 14,171 residents of the study area. Thus the average number of visits during the study period among those who ever visited the ED was 1.15, in other words 15% of the visits were repeat visits. The main analyses were constructed to compare the 14,171 first injury records identified as the subject's first attendance at ED for a home injury during the study period with those of the remaining 98,077 subjects. This has the effect of identifying all subjects who had one or more ED attendance for home injury during the study period.

The other datasets to be linked were the population distribution by housing group, age and sex (112,248 subjects, no missing data) and a file listing 54,913 properties, of which we restrict attention to the 54,801 with complete data. Table 1 shows how the three sets of data include different subsets of the variables. It is not possible to construct a comprehensively linked dataset at individual level enabling comparison of 14,171 injured and 98,077 uninjured subjects, because individuals in the latter group lack deprivation and proximity data.

**Table 1:** Variables included in the 3 datasets to be linked.

Variable	Included in dataset for		
	
	Injuries	Properties	Population
Housing type (build type, period, floor area)	Y	Y	Y
Sex	Y	N	Y
Age	Y	N	Y
Townsend score	Y	Y	N
Proximity to hospital	Y	Y	N
Type of injury (3 variables)	Y	N	N
Anatomical site of injury (3 variables)	Y	N	N

Following discussion of preliminary results it was decided to use actual deprivation and proximity scores for the injured, and to impute values randomly according to property type for the uninjured. This is appropriate because risk scores for the remaining variables, age and sex taken together as a forty two category categorical variable, were uncorrelated with risk scores for all other variables (see later), hence imputing according to property type alone and disregarding age and sex is a reasonable strategy.

The process of randomly imputing property records, and hence Townsend and proximity scores, to the 112,248 population according to housing type is not trivial. Using ordinary stratified sampling does not work, simply because the number of residents is larger than the number of properties. The overall occupancy ratio was 112,248 residents in 54,801 properties, i.e. 2.05 individuals per property, but this figure varied widely between the 94 property types. For example, for property type 1 there were just under 2 people per property, 114 properties and 226 population. We then choose randomly 114 of the 226 population to match one-to-one to the 114 properties in a random order, leaving the remaining 112 to be matched to a random sample of the 114 properties also in a random order. This simply achieves a maximal degree of representativity with an appropriate degree of randomness. A multi-stage randomisation and linkage process (further details available from the authors) successfully linked the vast majority (14,081/14,171) of first attendances with the merged population-properties file. This has the effect of producing a merged data file in which the deprivation and distance scores randomly imputed to the uninjured both incorporate the correct means to produce an appropriate degree of adjustment for confounding, and also the correct amount of variation to produce appropriate logistic regression coefficients to perform the adjustment.

The resulting linked file, comprising reconstructed data for all 112,248 residents, containing elements originating in population, properties and events files, has complete data for demographics, property type and randomly imputed Townsend and proximity measures. Among the 112,248 subjects, 14,081 are identified as having had one or more attendances for injuries following home accidents. Actual Townsend and proximity measures and the code for injury type 1 are available for each of the 14,081 injured individuals.

The actual and imputed Townsend and proximity values were summarised for the injured and uninjured and compared. This was done by t-test, paired or unpaired as appropriate, rather than by non-parametric methods which were associated with a serious loss of power due to the gross discreteness of the small area based, randomly imputed Townsend score. Associations between random and actual Townsend and proximity scores were characterised by Spearman rank correlations. Examination of these results, together with the low correlations of a risk score for age and sex with those for property type variables, Townsend and proximity, suggested it was appropriate to include in the logistic regression model composite Townsend and proximity scores, defined as the actual value in the injured and the randomly imputed value in the uninjured. Property size, age and build type were entered as categorical variables. Preliminary analyses indicated that Townsend score should be included as a continuous variable, but distance from hospital discretised into five categories. On account of the known marked sex-age interaction, sex and subject's age were entered together as a 42-group categorical variable, age being discretised into groups under 1, 1–4, 5–9, ..., 90–94 and 95 and over.

The main analysis proceeded as above, using the 14,081 who were ever injured during the study period as the group with the outcome of interest. Both univariate analyses for each explanatory variable in turn and a multivariate analysis were produced.

Also, the main analysis was repeated after re-running the randomisation parts of the linkage process. This step is more radical than might appear. In particular, due to the small amount of missing data, the randomised matching program does not pick up exactly the same set of 14,081 events records on both occasions. Essentially, the process draws 14,081 out of 14,114 potentially matchable events.

## Results

Table 2 shows summary statistics for actual and randomly imputed Townsend score and proximity. The summary statistics for the randomly imputed Townsend and proximity scores based on all 112,248 subjects are very similar but not identical to those for the 54,801 properties, from which they have been drawn. The mean randomly imputed Townsend score for the injured is very similar to the mean actual score in the 14,081 injured subjects, 0.85 (p = 0.78). Conversely, the randomly imputed distance measures are similar in injured and uninjured (p = 0.12), but the randomly imputed values are significantly greater than the actual ones in the 14,081 injured (p < 0.001). For the Townsend score, the randomly imputed scores are highly significantly higher (i.e. more deprived) for the injured (mean 0.86) than the uninjured (mean 0.70, p < 0.001). For Townsend score and distance alike, the difference between the actual mean in the injured and the mean of randomly imputed values in those not injured is approximately correct to adjust for in the subsequent multivariate analysis, and the process incorporates the appropriate degree of variation at individual level.

**Table 2:** Summary statistics (mean and SD) for actual and randomly imputed Townsend score and proximity to hospital.

Basis	Series	n	Townsend score	Distance (km)
All properties		54,801	+0.74 (2.75)	8.33 (5.06)
Randomly imputed	All	112,248	+0.72 (2.77)	8.36 (5.05)
	Not injured	98,167	+0.70 (2.77)	8.37 (5.06)
	Injured	14,081	+0.86 (2.76)	8.30 (5.03)
Actual	Injured	14,081	+0.85 (2.77)	7.73 (4.87)

Table 3 gives Spearman rank correlations between random and actual Townsend and proximity measures. While all of these are statistically significant (p < 0.001), most are quite small. The correlation of nearly 0.2 between random and actual Townsend scores reflects the unsurprising, substantial variation in Townsend score between property types.

**Table 3:** Spearman rank correlations between random and actual Townsend and proximity measures.

Within groups between variables	N	Spearman rank correlation	95% confidence interval
Townsend v. distance			
Randomly imputed	112,248	0.107	0.101 to 0.113
Actual	14,081	0.051	0.034 to 0.067
Randomly imputed v. actual			
Townsend	14,081	0.193	0.177 to 0.209
Distance to ED	14,081	0.055	0.039 to 0.072

The effect of the random imputation process was explored in the main logistic regression model. In the final model the effect of age and sex jointly was dominant, followed by build type, then distance, property age, Townsend score (all p < 0.001) and floor area (p = 0.007). Table 4 shows the univariate and adjusted results with regard to build type, the housing variable with the clearest association with the proportion of subjects who ever attended for a home injury. Adjustment for the confounding effects of the other variables made some difference to the odds ratios, but the doubled risk of injury in build type D remained essentially unaltered.

**Table 4:** Main logistic regression model results. All first injuries (14,081 subjects out of 112,248). Odds ratios and X^2 ^tests for effect of build type on proportion of subjects ever injured (a) unadjusted; (b) adjusted for other factors after random imputation of deprivation and distance scores to the uninjured; and (c) adjusted for other factors after re-randomisation.

Build type	Number of residents	Univariate model	Adjusted for other factors, original random imputation	Adjusted for other factors, re-randomisation
		
		Odds ratio	Odds ratio (95% CI)	Odds ratio (95% CI)
A	15,877	0.790 (0.746–0.837)	0.890 (0.831–0.954)	0.892 (0.832–0.955)
B	35,791	1.049 (1.009–1.091)	1.108 (1.055–1.165)	1.109 (1.055–1.166)
C	280	1.003 (0.703–1.431)	1.106 (0.768–1.592)	1.113 (0.774–1.600)
D	2,695	2.046 (1.863–2.247)	2.074 (1.870–2.301)	2.074 (1.869–2.301)
E	57,605	1.000	1.000	1.000
X^2 ^(4 df)		327.5	254.5	254.1
p-value		<0.001	<0.001	<0.001
Distance to ED (km)				
4.26 and below		1.431 (1.352–1.514)	1.469 (1.384–1.560)	1.467 (1.381–1.557)
4.27 – 5.57		1.288 (1.211–1.369)	1.413 (1.317–1.516)	1.409 (1.314–1.512)
5.58 – 8.69		1.385 (1.308–1.467)	1.356 (1.276–1.440)	1.352 (1.273–1.437)
8.70 – 13.25		1.168 (1.100–1.240)	1.243 (1.166–1.325)	1.248 (1.170–1.330)
13.26 and above		1.000	1.000	1.000
X^2 ^(4 df)		194.8 (p < 0.001)	179.9 (p < 0.001)	176.9 (p < 0.001)
Townsend score		1.020 (1.014–1.027)	1.016 (1.008–1.024)	1.016 (1.008–1.024)
X^2 ^(1 df)		36.8 (p < 0.001)	15.4 (p < .0.01)	15.9 (p < 0.01)

The results of the main multiple logistic regression model in table 4 were used to construct risk scores for each individual representing age and sex, the three property variables, and the composite distance measure. Table 5 shows parametric correlations of the risk score for sex and age with those for the three property variables and the composite distance measure, and the composite Townsend score. (This is, of course, equivalent to using a risk score based on it as it is entered as a linear factor in the model). Even though all but one of these correlations is highly significant, all of them are sufficiently small that we can regard the random imputation process as reasonable.

**Table 5:** Parametric correlations of risk scores for age and sex with those for other factors.

Parametric correlation of risk score for age and sex with:		95% confidence interval
Risk score for floor area	+0.027	+0.021 to +0.033
Risk score for age of property	-0.020	-0.026 to -0.014
Risk score for type of property	+0.028	+0.022 to +0.034
Townsend score working	+0.026	+0.020 to +0.031
Risk score for distance	-0.003	-0.009 to +0.003

## Discussion

The main methodological finding of the study is that the random imputation process developed here is a reasonable one. This approach enabled us to base analyses on a very large dataset notwithstanding confidentiality issues precluding comprehensive linkage directly at individual level. It is feasible to incorporate randomisation into the linkage process, even when the target group is larger than the source. The reasonableness of the imputation process can be judged by comparison of the actual and imputed variables for the injured population. For the Townsend score the actual and imputed scores are essentially identical in distribution, thus showing that the methodology does not produce a biased result. For the distance variable the mean imputed value is 7.4% higher than the actual distance. Whilst this difference is statistically significant the magnitude of the effect on residual confounding cannot be large, given the odds ratios for attendance rates by distance in Table 4, which indicate that a 1% change in distance produces around a 1% change in the odds ratio for attendance.

The low correlation between randomly imputed and actual values, for distance from hospital and Townsend score, could result in attenuated regression coefficients and hence underadjustment for the confounding effect of these variables. For distance, which is by far the more influential of the two variables, a logistic regression in which the entire population of 112,248 is assumed to have the same distribution into the 5 distance groups as applies to the 54,801 properties gives odds ratios 1.469, 1.318, 1.445, 1.200 for the first 4 distance categories relative to the 5^th ^(most distant) one. These figures are similar to those obtained in the univariate logistic regression based on the composite distance measure, and suggest that the latter regression coefficients, and hence also those in the multiple regression, may be attenuated by around 10% only.

Of primary importance to the validity of the methodology set out here, the results obtained after a second randomisation were almost identical. The unadjusted analyses for age-sex and housing type were unaltered, as these variables do not come from the random imputation. The analyses for deprivation and proximity and the results of multivariate analyses for build type and other variables were altered, but only to a very minor degree. These results provide considerable reassurance that the random element that was necessary in order to achieve the linkage process introduced very little additional uncertainty into the final analyses.

It appears that injured people tend to live in property types more associated with deprivation than the uninjured. Their actual Townsend scores are in line with what we would expect from their property types. On the composite data, i.e. when we replace random by actual Townsend scores for the injured only, there is a substantial difference in mean Townsend score, 0.85 v. 0.70, and all the 0.15 points difference is attributable to a real effect of deprivation on risk.

Conversely, the injured and uninjured tend to live in property types equally distanced from hospital. The actual distance is less for the injured than the randomly imputed distance, which is in line with the known tendency for hospital attendance for less serious types of injury to be related to proximity^(6)^. On the composite data (with means 7.73 v. 8.37 km), nearly the whole of the difference (0.57 out of 0.64 km) is attributable to this self-selection effect.

## Conclusions

This process is an important methodological development to increase the power of linkage studies when all individual data elements are not available for all individuals. As a result the analysis was based on 112,248 subjects and not on ninety-four groups. Thus, the power to detect important differences is substantially enhanced.

Further work is continuing in the relationship between specific features of built type and injury occurrence, using the methodology described in this paper.

## Competing interests

The author(s) declare that they have no competing interests.

## Authors' contributions

All authors contributed to the design of the study. RGN devised the conceptual design of the data imputation and carried out the statistical analysis. All authors read and approved the final manuscript.

## Pre-publication history

The pre-publication history for this paper can be accessed here:


